# Exploring the Impact of Hyaluronic Acid Addition Order on the Structural Integrity and Quality of Myofibrillar Protein Gels

**DOI:** 10.3390/molecules31060923

**Published:** 2026-03-10

**Authors:** Sahar Mehraban, Anna Stępień, Marzena Zając

**Affiliations:** 1 Department of Animal Product Technology, Faculty of Food Technology, University of Agriculture, Balicka 122, 30-149 Kraków, Poland; sahar.mehraban@student.urk.edu.pl; 2 Department of Engineering and Machinery for Food Industry, Faculty of Food Technology, University of Agriculture, Balicka 122, 30-149 Kraków, Poland; anna.stepien@urk.edu.pl

**Keywords:** hyaluronic acid, meat protein gels, food additive, physical properties, rheology

## Abstract

In this study, we investigated hyaluronic acid (HA) as a functional biopolymer for improving the processing performance of myofibrillar protein (MP) gels. Our focus was on the order of incorporation and concentration of HA as controllable process parameters, and their effects on water-holding capacity, rheological behaviour, texture, colour and microstructure of MP gels. The experimental results demonstrated that HA promoted the formation of a denser and more homogeneous protein network, as confirmed by microstructural analysis and significantly enhanced water retention. From a mechanical perspective, HA incorporation decreased hardness and chewiness while increasing adhesiveness, thereby improving overall gel functionality. Importantly, the simultaneous dissolution of HA with meat and water produced superior outcomes compared to post-addition, highlighting the role of ingredient addition sequence as a relevant process design factor. The slight colour variations remained within acceptable quality limits. Our findings provide new insights into protein hydrocolloid interactions in gel systems and indicate how HA can be strategically integrated into processing operations to improve product yield, quality and consumer acceptance in the meat industry.

## 1. Introduction

The growing demand for high-quality meat products has increased interest in functional ingredients capable of improving both sensory and technological characteristics. Among these, hyaluronic acid (HA)—a naturally occurring glycosaminoglycan—has attracted considerable attention for its exceptional water-binding capacity and viscoelastic properties [1]. In biological systems, HA plays an essential role as a lubricant, structural stabiliser, space filler and shock absorber, particularly in the joints, skin and cartilage [2,3]. Owing to its moisture retention capacity, mechanical resilience, lack of immunogenicity and non-toxicity, HA has emerged as an attractive biomaterial for various applications, including food systems [4,5]. Furthermore, HA is considered a prebiotic and has been associated with multiple health benefits, such as anti-inflammatory, antioxidant, anti-ageing and angiogenic effects [6,7,8].

The application of HA in foods has expanded following regulatory approvals in several regions, including Japan, South Korea, China and the European Union, while in countries such as the USA, Canada, Italy and Belgium, it is recommended as a dietary supplement [9,10]. HA is also a natural component of the human body [11]. Nevertheless, its rapid degradation, limited stability and inability to form self-supporting aggregates in aqueous environments pose challenges for its direct application in food matrices [12]. In previous studies, it has been demonstrated that intermolecular hydrogen bonding, as well as electrostatic and van der Waals interactions between HA and proteins, can enhance stability and functional performance, thereby improving suitability for food applications [13,14].

In comminuted meat products—such as sausages and patties, which are typically formulated as oil-in-water emulsions—myofibrillar proteins (MP) play a key role in stabilising the protein network responsible for texture, water-holding capacity and product yield. In recent studies, it has been shown that HA can improve MP emulsifying efficiency, enhance water retention and reduce cooking losses. However, excessive HA addition may disrupt the gel network and lead to undesirable textural changes, underscoring the importance of optimising both HA concentration and incorporation strategy in meat formulations [15].

From a food engineering perspective, the timing of HA incorporation represents a controllable process variable that influences protein network formation, water diffusion and gel mechanical strength [16]. Despite the promising functionality of HA, systematic studies, in which the effects of HA concentration and incorporation sequence on microstructure development and gel functionality are simultaneously addressed, still remain limited. Addressing this gap is essential to translating laboratory-scale findings into scalable, industrially applicable processes [17,18,19].

Although HA has traditionally been used for cosmetic and pharmaceutical applications, its potential in food technology has become increasingly evident. HA has been investigated in various food matrices, including yoghurt [8,20], kefir [21], milk [22], skimmed milk [23], corn starch [24], homogenised sausage [25], and beef, where it improved oxidative stability and sensory quality during refrigerated storage [26]. HA’s water-holding capacity, stability and ability to modify texture have been demonstrated in these studies; however, challenges related to optimal dosage and processing conditions have also been reported. For example, Zając et al. [25] observed that low HA concentrations did not consistently reduce cooking losses in meat emulsions, whereas excessive HA addition could negatively affect the gel structure. Similarly, investigations in dairy and starch systems have shown that HA can influence gelation behaviour, thermal stability and rheological properties depending on formulation and processing conditions [20,21,22,23,24].

Therefore, in the present study, a systematic investigation is conducted regarding the effects of HA concentration, water content and the order of HA incorporation on the rheological, thermal, structural, physical and textural properties of MP gels. By clarifying the combined influence of formulation and processing variables, this work is aimed at optimising gel functionality and contributing to the development of higher-quality meat products with improved technological performance and consumer acceptability.

## 2. Results and Discussion

### 2.1. Rheological Characteristics

**Figure 1:**
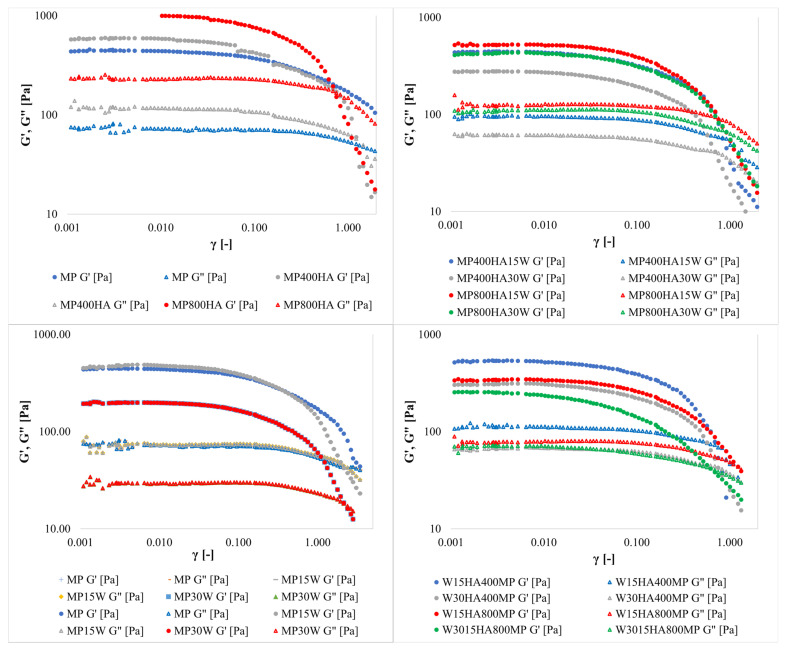
Storage and loss modulus values as function of strain amplitude at constant oscillation frequency (1 Hz) for myofibrillar protein gels at 20 °C.

The strain sweep test was used to examine linear and non-linear viscoelastic regions of protein gels. The results of the test are shown in Figure 1. In lower strain conditions, the storage modulus for all samples was significantly higher than the loss modulus, which indicates the solid-like elastic nature of the investigated materials. HA incorporation increased storage modulus (G′) and loss modulus (G″) reflected altered viscoelastic behaviour and improved functional properties, although the gels were softer. G′ and G″ values increased markedly with the addition of HA, particularly at higher concentrations (800 mg/100 g MP). The difference between G′ and G″ curves increased with HA addition, indicating stronger viscoelastic interactions, while the gel hardness decreased. The denser and more flexible structure of gels with HA indicated strong protein-polysaccharide molecular interactions. The large number of hydroxyl groups in HA, which act as hydrogen bond donors, results in the multiplication of active sites and promotes cross-linking of the components. A similar observation was reported for ginkgo seed protein gels prepared with HA [27]. In contrast to HA, the addition of water to the systems negatively influenced the elasticity of the gels. Wang et al. [28] employed oscillatory rheology, SDS-PAGE and SEM to comprehensively analyse the gel properties of MP from various meat sources, including pork. Their findings revealed that pork MP exhibited the highest G′ and loss G″ moduli, indicating a stronger gel network. This was further supported by SDS-PAGE results, which showed that pork MP had the highest myosin heavy chain (MHC) content, a key protein contributing to gel strength. Additionally, the yield stress values exhibited a notable rise, suggesting improved gel stability and viscoelastic behaviour.

These findings are consistent with those noted in the work by Zając et al. [25], who also reported improved functional elasticity and reduced syneresis in meat emulsions with the incorporation of HA. In the present study, the enhanced G′ and G″ values observed upon HA incorporation can be attributed to the ability of HA to form hydrogen bonds and electrostatic interactions with MP, thereby reinforcing the gel matrix. Furthermore, the superior water-holding capacity of HA plays a crucial role in minimising syneresis and maintaining the desired texture of meat products. This is particularly beneficial for meat formulations in which water retention is critical for quality. The combined effect of HA’s interaction with proteins and its water-holding capacity contributes to the overall improvement in rheological properties and texture of the MP gels. In previous studies, it has been shown that hydrocolloids, such as carrageenan and xanthan gum, enhance gel elasticity. Nanta et al. [29] observed improved viscoelastic properties in soy protein isolate meat analogues with these hydrocolloids. Similarly, Dick et al. [30] highlighted the role of hydrocolloids in enhancing the rheological and textural properties of 3D-printed meat products. These findings indicate a consistent trend of improved gel elasticity across various hydrocolloids.

While various hydrocolloids have demonstrated beneficial effects on elasticity, HA distinguishes itself by its remarkable water-holding capacity, making it particularly beneficial in meat formulations. Overall, the results suggest that HA not only enhances the rheological properties of MP gels but also offers unique advantages in terms of water retention, which is crucial for maintaining the quality and texture of meat products.

Rheological measurements indicated that HA-containing gels had a more cohesive but softer network. Such an enhancement is seemingly attributable to the negative charge of HA’s carboxyl groups and its intermolecular hydrogen bonds, which jointly endow it with large hydrodynamic volume and distinctive viscoelasticity. These are among the properties making HA a promising candidate in both biomedical and nutritional applications [31,32]. The increases in G′ and G″ observed in this study can probably be attributed to HA’s capacity to establish hydrogen bonds and electrostatic interactions with MP, thus forming a more cohesive and functionally effective network.

To further understand the viscoelastic behaviour of the gels, the crossover point where the G′ and G″ are equal (G′ = G″) signifies a transition in the material’s rheological behaviour. At this point, the material shifts from a predominantly elastic (solid-like) state to a more viscous (liquid-like) one. This transition is often referred to as the flow point, indicating the onset of flow behaviour, or the gel point in cases where the material undergoes gelation. Almost all the tested samples demonstrated a flow point at γ values varying around 1. Only the MP sample intersection of the curves was noted beyond the investigated range.

**Figure 2:**
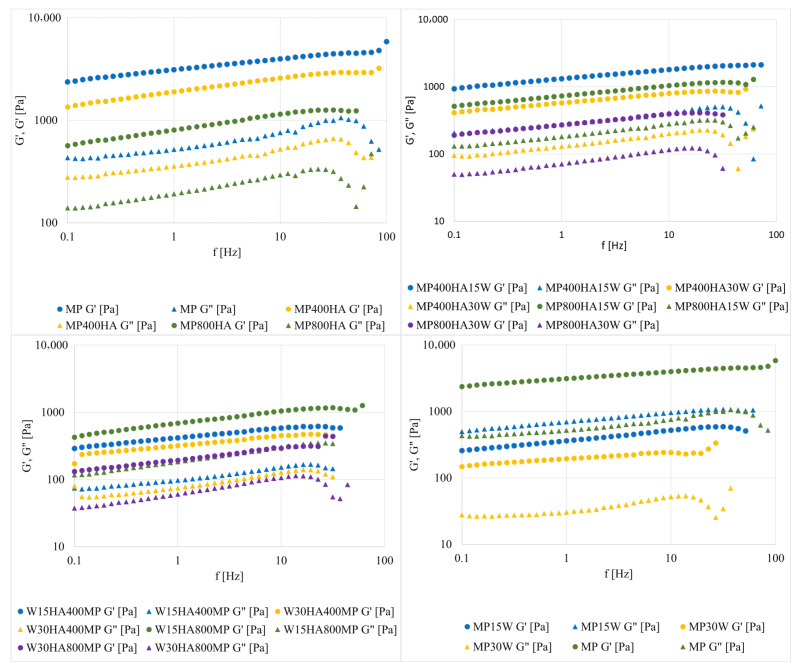
Frequency sweep data for myofibrillar protein gels with HA at 20 °C.

In Figure 2, the storage and loss modulus values are presented as a function of oscillation frequency determined for protein—HA systems. Higher G′ than G″ values for the whole tested range proved a stable structure of the samples. All curves indicated that the dynamic moduli remained largely independent of the applied frequencies. This behaviour is primarily attributed to the measurements being conducted at room temperature, which is significantly lower than that for gelation. In these conditions, the material’s structure remains stable and influenced by its thermal history, allowing G′ and G″ to reach values of equilibrium [33].

These findings are supported by those achieved in recent studies conducted in plant-based systems, where HA significantly improved gelation and mechanical characteristics through non-covalent interactions and network densification. To demonstrate this, Cheng et al. [34] showed that variations in HA’s molecular weight and pre-dissolution states can have an impact on its entrapment in protein matrices, thereby influencing the stability and spatial distribution of protein gels derived from ginkgo seeds. Lin et al. [35] reported that HA-casein complexes also enhanced foam structure and elasticity. This suggests that the advantageous effects observed in our meat system may also apply to other protein matrices with different structural characteristics.

### 2.2. Thermal Analysis via Differential Scanning Calorimetry

The results of Differential Scanning Calorimetry (DSC) further confirmed that HA influenced the thermal properties of MP gels in a formulation-dependent manner, with both increases and decreases in ΔH and Tp observed. As shown in Table 1, the effect of HA on thermal stability was formulation-dependent, with certain treatments showing increased denaturation temperatures, whereas others exhibited reductions, suggesting complex interactions among HA, water distribution and the protein network. The enthalpy change (ΔH) values also varied significantly among treatments (*p* < 0.05), indicating that HA influenced heat resistance and water-binding behaviour depending on formulation conditions.

These effects may be attributed to interactions between HA and the hydrophilic regions of the protein, which can influence water distribution and structural transitions during heating. A similar effect has been observed for HA-enriched dairy products, in which HA improved the integrity of the protein matrix and enhanced heat resistance [22]. This finding suggests that the functional role of HA in meat gels may extend to other protein-based food systems, highlighting its potential as a versatile ingredient in food formulations.

Consistent with our observations, D. Wang et al. [36] demonstrated that thermal treatment promotes the interaction between sodium hyaluronate (SH) and whey protein isolate/hydrolysate (WPI/WPH), leading to enhanced thermal stability. Specifically, they found that the formation of WPI-SH and WPH-SH complexes improved the denaturation temperature of the proteins. This supports the hypothesis that HA, through similar interactions with MP as in our MP gels, can enhance thermal resilience.

In previous research, it has also been shown that the addition of hydrocolloids influences the denaturation enthalpy (ΔH) of proteins, indicating changes in water-binding capacity and thermal resistance. For instance, Nanta et al. [29] noted that hydrocolloids can significantly affect the ΔH of soy protein isolates.

Overall, these results underscore the significance of HA in influencing the thermal properties of protein gels, thereby affecting their response to heat stress and overall functional quality in various food applications.

### 2.3. Determination of MP-HA Gels’ Physical Properties

#### 2.3.1. Cooking Yield and pH Analysis

The average pH of all samples was 6.9, showing no significant differences between them (Table 2). The interaction between HA and MP may have a buffering effect, aiding the maintenance of the protein structure’s stability during cooking. These results suggest that HA may interact with the MP and possibly affect the surface charge of the protein molecules. However, these changes were within the acceptable range and did not noticeably influence the overall acidity of the protein matrix.

In Table 2, the mean yields per group are shown, along with standard errors. The statistical analysis indicated a significant difference only between the W15HA800MP (82.3%) and MP30W (61.2%) groups (*p* = 0.045).

Groups, such as MP800HA, MP800HA15W and W30HA800MP, exhibited relatively high yield values comparable to W15HA800MP, but without significant statistical differences. Descriptive statistics show that the highest mean yield was observed in W15HA800MP (82.28%), followed by MP800HA (81.28%) and MP800HA15W (80.87%), while MP30W (61.15%) exhibited the lowest yields. Despite these numerical differences, the majority of treatments showed no statistically significant differences between one another. In previous research on MP and HA [15], it was noted that increasing the HA concentration significantly increased cooking loss, despite the well-known water-holding capacity of HA. These changes were observed at 179 mg of HA per 100 g of MP, which is considerably lower than the amounts used in our experiment (400 and 800 mg). However, the HA used by Zając et al. [15] was characterised by a much higher molecular weight, which may have influenced the results.

#### 2.3.2. Colour Determination

Colour determination, as detailed in Section 3, was performed to measure colour parameters such as L*, a*, b*, whiteness index (WI) and yellowness index (YI) in the cooked MP gels with varying concentrations of HA and water levels. A summary of the results, along with the corresponding statistical analysis, is presented in Table 3.

The L values—representing brightness—ranged from 73.89 to 74.91, and no significant differences were found among them. Slight variations in a* (red-green) and b* (yellow-blue) were observed, with more negative a* values at higher HA concentrations, reflecting a minor shift towards green. However, these changes were small and remained close to the control, suggesting that the visual quality of MP gels was not compromised. Considering that these results are based on MP gels rather than whole meat, the observed colour variations were minimal and fell within an acceptable range for model meat systems. To evaluate the practical impact of HA on colour, all samples were compared with the control (without HA) by calculating ΔE. According to CIELAB, ΔE values below 1 indicate that a standard observer would not perceive a colour difference [37]. This positive result aligns with previous studies in which the minimal impact of hydrocolloids on meat colour has been highlighted [29,36].

The a* values—indicating the red-green spectrum—varied significantly among the samples, ranging from −1.78 (MP800HA30W) to −1.51 (MP15W). The MP800HA30W sample exhibited the most negative a* value of −1.78, indicating a stronger shift towards the green spectrum. In contrast, the MP400HA15W sample had a slightly less negative value of −1.71, appearing somewhat less green. Other samples, such as MP15W (−1.51), showed even less green hue, further illustrating the range of colour variations among the products.

A significant decrease in a* value was observed in MP800HA30W compared to MP400HA15W, underlining the effect of HA concentration on red-green colour balance. Similarly, Kołodziejczak et al. [38] found that increasing HA concentrations in plant-based gels significantly reduced a* values, enhancing the green hue of the samples.

The b* values ranged from 5.21 (MP400HA30W) to 5.96 (MP400HA), with MP400HA showing the most intense yellow hue. Significant differences (*p* < 0.05) were detected, particularly between MP400HA and MP400HA30W, indicating that HA concentration influences the yellow-blue balance. The combination of higher HA levels with water (MP400HA30W) led to a decrease in b* values, indicating a more balanced yellow-blue tone.

In addition to the conventional L*, a* and b* colour parameters, we measured WI and YI to provide a more complete image of the gel’s appearance. WI captures overall brightness and whiteness, while YI reflects subtle shifts in yellow tones, which can indicate minor compositional or processing-related changes that may not be evident in the case of L*, a* and b* values alone. The WI values ranged from 73.26 to 74.32, with no significant differences observed among the samples (*p* > 0.05), indicating comparable levels of whiteness. Meanwhile, YI ranged from 9.94 (W30HA800MP) to 11.45 (MP400HA), with the latter exhibiting the highest yellowness. The significant differences (*p* < 0.05) in YI values indicate that HA concentration affects the yellow appearance of the gels.

While HA had no significant effect on lightness, it notably influenced the red-green and yellow-blue balances. Specifically, the combination of MP400HA and 15 mL water (MP400HA15W) resulted in a marked increase in both a* and YI values (*p* = 3.99 × 10^−6^), leading to a more pronounced red and yellow appearance. Colour stability is a crucial factor for consumer preference, and the minimal changes observed in the colour of the meat gels suggest that HA does not negatively impact their visual appeal. This characteristic is particularly advantageous compared to other hydrocolloids, such as soy protein or modified starches, which can sometimes induce undesirable colour shifts in meat products [39].

Although our study was focused on MP gels, while Wang et al. [40] investigated whole pork gels under high-pressure treatment, comparable trends in lightness (L*) were observed. In both studies, it was shown that the addition of functional ingredients (HA in our study, NaCl/SPP in Wang et al.) had minimal effects on L*, while influencing a* and b* values. These findings support the idea that hydrocolloid-type additives can modulate meat gel colour without significantly compromising brightness, and are consistent with previous research on meat colour stability, in which the role of myoglobin state, pH, oxidation and processing conditions has been emphasised in determining instrumental colour parameters such as L*, a* and b* [41].

These findings are consistent with recent advances in meat colour research, in which it is indicated that factors such as myoglobin state, pH, lipid oxidation, processing conditions and instrumental measurement protocols can influence L*, a* and b* values in meat products [42]. This supports our observation that the addition of HA to MP gels did not significantly affect L*, while slight variations in a* and b* are expected due to intrinsic and processing-related factors. However, the addition of HA caused slight changes in a* and b* values, while L* and WI remained largely unchanged. This suggests that the observed colour differences are minor and unlikely to be noticed by consumers, meaning that the visual appeal of MP gels is maintained despite HA incorporation.

Maintaining an attractive colour is essential for consumer acceptance, as it often serves as an indicator of freshness and quality [40]. The pH analysis also showed that adding HA did not significantly change the pH levels of the meat gels, supporting the idea that HA can be used without compromising the overall quality of the product [1,40]. Due to the fact that HA is stable with regard to both colour and pH, it has significant potential as a functional ingredient in meat formulations. It can improve texture and water retention, while preserving important visual and sensory qualities for consumers.

Overall, these results indicate that HA can be incorporated into MP gels without compromising visual quality, supporting its potential application in meat products from a consumer acceptability perspective.

#### 2.3.3. Texture Properties

In this study, the textural properties of MP gels were analysed, focusing on key parameters such as hardness, adhesiveness, springiness, cohesiveness, chewiness and resilience. The results (Table 4) clearly demonstrate the significant impact of HA and water content on the texture of these meat gels.

As shown in Table 4, we observed a clear trend of decreasing hardness with increasing water content, particularly in samples with higher HA concentrations. The control sample (MP) exhibited the greatest hardness (23.26 ± 1.90), while the MP800HA30W sample, which contained the most HA and water, displayed the lowest hardness values (3.49 ± 0.53). This suggests that the combination of HA and water disrupts the protein network, leading to a softer texture, yet a more hydrated and flexible network induced by HA and water, rather than structural weakening. This finding is consistent with that noted in the work by Zajac et al. [25], who reported that incorporating HA softens meat gels by enhancing water retention and modifying the protein matrix.

We observed a significant increase in adhesiveness with the addition of HA, particularly in the MP800HA15W and W30HA800MP samples. For instance, the W30HA800MP sample showed an adhesiveness value of −65.88 ± 11.03, indicating a much stickier texture compared to the control. This increase in stickiness can be attributed to HA’s hydrophilic nature, which enhances water binding and increases the surface stickiness of the gel. Bao et al. [39] also reported that polysaccharides such as HA improve the adhesiveness of meat products by enhancing water binding.

In addition to these textural changes, rheological measurements further confirmed the impact of HA on texture. The addition of HA significantly increased the storage modulus (G′) and loss modulus (G″) of the samples, particularly in those with higher HA concentrations. This suggests that HA modifies the gel network, enhancing cohesiveness and water retention, while reducing hardness and chewiness. The increase in adhesiveness, coupled with these rheological changes, indicates that HA not only improves the stickiness of the texture but also contributes to a more stable and cohesive gel structure. These findings align with those achieved in previous research by Bao et al. [39], who highlighted the role of HA in improving both texture and rheological properties.

The springiness remained relatively stable across the majority of samples. The MP and MP15W samples exhibited the highest springiness values (0.91 ± 0.02 and 0.87 ± 0.03, respectively), while samples with higher HA concentrations showed slightly less springiness. This suggests that, although HA affects other textural properties, it does not significantly alter the gel’s ability to recover from deformation. This finding aligns with that reached by Wang et al. [28], who noted that springiness is less affected by HA addition compared to other textural parameters.

The cohesiveness values were comparable between MP (0.55 ± 0.05), MP400HA (0.53 ± 0.02) and other samples, with no statistically significant differences observed (*p* > 0.05). However, the lowest cohesiveness values were noted in MP800HA15W (0.34 ± 0.03) and W30HA400MP (0.43 ± 0.11), indicating weaker protein–protein interactions in these samples.

This decrease in cohesiveness can be attributed to the higher HA content, which disrupts the protein matrix and weakens internal bonds. Similar observations were made by Nanta et al. [29] in research involving hydrocolloids.

Chewiness–a parameter that combines both hardness and cohesiveness–significantly decreased in the MP800HA30W sample (0.82 ± 0.13), indicating a notable softening effect as a result of adding both HA and water. The control sample (MP) exhibited the highest chewiness value (11.58 ± 1.26), further supporting the notion that HA causes its reduction not by weakening the protein network, but by promoting the formation of a more hydrated and flexible protein–polysaccharide matrix. This reduction is consistent with that observed in a previous study by Zajac et al. [25], demonstrating that HA softens meat products, making them less chewy and easier to consume.

Resilience was similar across all samples (*p* > 0.05), indicating that HA—either alone or with water—does not significantly impact the gel’s recovery from compression. In contrast, Nanta et al. [29] noted that hydrocolloids reduced resilience in meat analogues. The discrepancy between these findings could be attributed to differences in experimental conditions, such as the types of hydrocolloids used, their concentrations or preparation methods.

Statistical analysis confirmed significant differences in the textural properties, with the most notable variations observed in hardness (F = 19.66, *p* = 2.73 × 10^−13^), adhesiveness (F = 7.90, *p* = 2.37 × 10^−7^) and chewiness (F = 18.34, *p* = 8.67 × 10^−13^). These findings underscore the significant role HA plays in altering the texture of meat gels.

The incorporation of HA into MP matrices modifies texture by producing softer but more cohesive gels with improved water retention of the gels. Our findings indicate that the order of incorporating HA into the MP matrix affects the final product quality; specifically, samples in which HA was first dissolved in water before being added to the MP exhibited distinct textural and rheological properties compared to those in which HA was directly mixed with MP and water. This suggests that the solubility and interaction of HA with the protein matrix can influence the gel’s mechanical properties and water-holding capacity. Furthermore, both the concentration of HA and the added amount of water play critical roles in determining the quality of the MP gels. Higher concentrations of HA (800 mg/100 g MP) combined with adequate water levels (30 mL) resulted in improved cooking yields and enhanced textural properties, such as reduced hardness and increased adhesiveness. This aligns with existing literature, in which the benefits of hydrocolloids in enhancing water retention and overall product quality are highlighted. The primary aim of this study was to investigate the effects of different concentrations and incorporation methods of HA on the rheological, thermal, physical and textural properties of MP gels. Throughout the analysis, it is evident that HA not only enhances the mechanical properties of the gels, but also contributes to improved water retention and visual appeal, making it a valuable ingredient in meat formulations. Future research should be focused on further exploring optimal conditions for HA incorporation to maximise its benefits in various meat formulations.

#### 2.3.4. Scanning Electron Microscopy (SEM) Analysis

**Figure 3:**
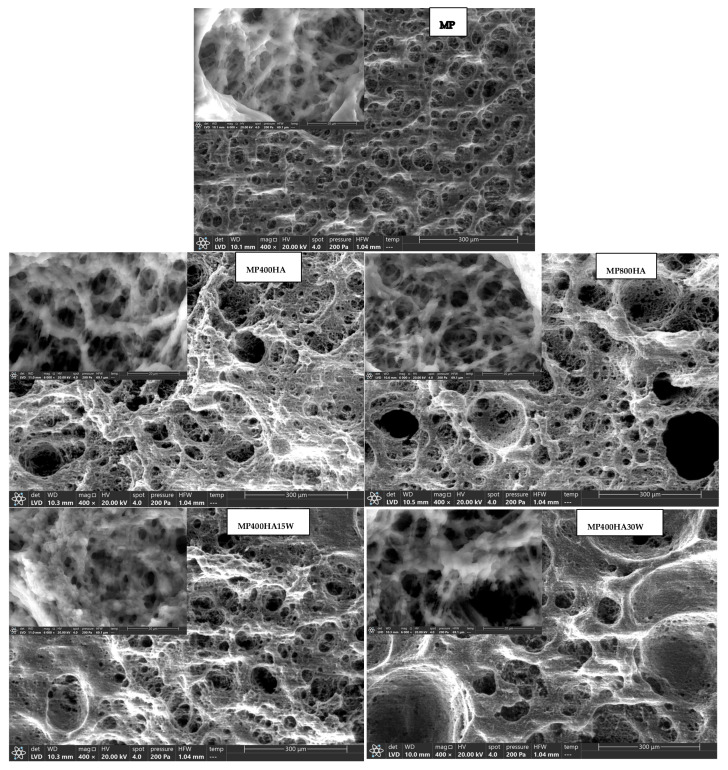

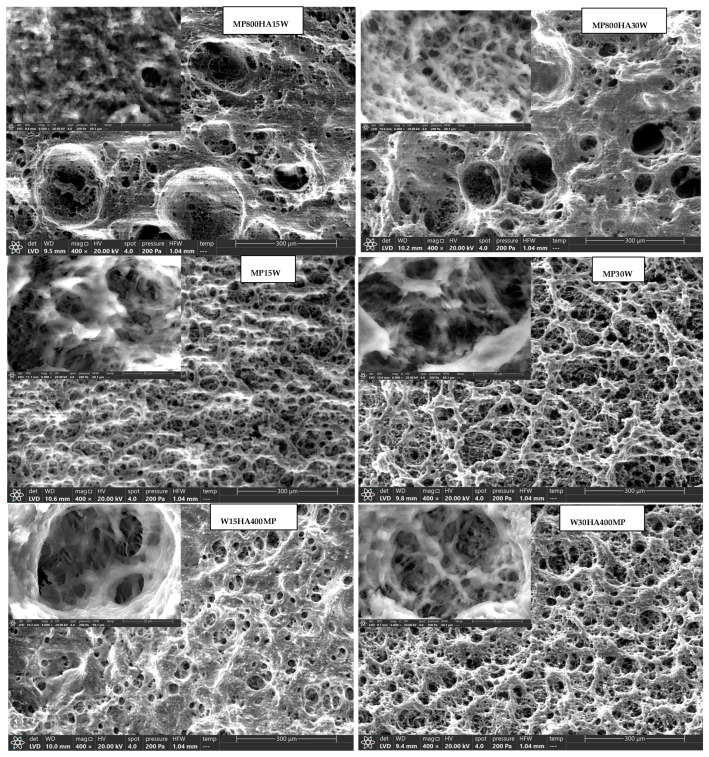

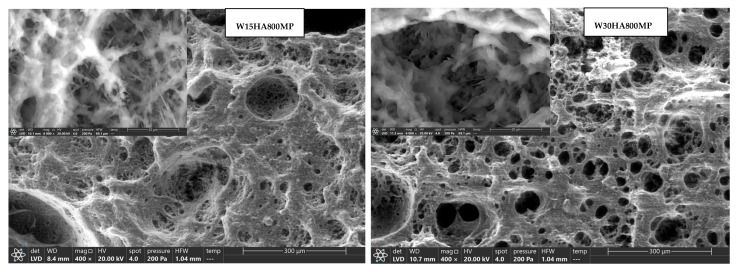
SEM micrographs showing microstructure of myofibrillar protein gels at different levels of hyaluronic acid (HA) and water. Numbers correspond to sample codes listed in Table 6. Magnification: 400×, Voltage: 20.0 kV.

Microstructural analysis using scanning electron microscopy (SEM) revealed distinct differences in the structural organisation of the protein gels (Figure 3). The control sample (MP) exhibited a coarse, porous structure with visible voids, suggesting weak water retention capabilities. These results are consistent with the findings obtained by Wang et al. [28], who also observed variations in the microstructure of MP gels from different meat sources using SEM. They reported that the microstructure of the gels significantly impacted their water-holding capacity and cooking loss. Similar findings were reported by Wu et al. [24], who noted that HA effectively reduced phase separation in hydrocolloid-stabilised food systems.

The SEM analysis provided valuable insights into the microstructural differences found in MP gels with varying concentrations of HA. The control sample (MP) displayed a coarse and porous structure with visible voids, signifying poor water retention properties. In contrast, HA-containing samples—particularly MP800HA30W–exhibited a dense and compact network with uniform protein distribution. Higher magnification images revealed that the MP strands in HA-treated samples were more tightly packed, forming a homogeneous and interconnected structure. This compact arrangement likely contributed to the enhanced water-holding capacity and reduced cooking loss observed in these samples. SEM images showed that HA-containing samples formed a more compact protein network compared to the control. Quantitative SEM image analysis (Table 5) confirmed that samples prepared via simultaneous addition, such as MP800HA30W, exhibited smaller average pore sizes and a more uniform pore distribution compared to sequentially added systems, supporting the formation of a denser and more homogeneous protein network. This structural difference can be attributed to the synchronised hydration of HA and unfolding of MP during thermal gelation, which promotes cooperative network formation. These findings are consistent with those achieved in earlier studies carried out by Wang et al. [28] and Wu et al. [24], who emphasised the significance of microstructure in determining the functional properties of protein gels. Overall, the results suggest that incorporating HA significantly improves the structural stability and functional properties of MP gels, making it a promising additive for enhancing the quality of meat products in food formulations.

This research offers strong proof that HA improves the rheological, structural and functional qualities of animal protein gels. The incorporation of HA significantly improved water-holding and gel network homogeneity, while thermal properties were formulation-dependent. These enhancements suggest that HA could serve as a valuable ingredient in meat products. The key findings demonstrate that HA contributes to increased cooking yields, reduced moisture loss and improved texture parameters, including reduced hardness and chewiness as well as increased adhesiveness and cohesiveness. Although the W15HA800MP sample exhibited the highest cooking yield, its value alone was not considered the sole criterion for determining the optimal formulation. Instead, a comprehensive evaluation integrating cooking yield, textural attributes, rheological behaviour, thermal stability and microstructural characteristics was applied. In this context, the MP800HA30W sample—prepared via simultaneous addition of MP, HA and water—demonstrated the most balanced and functionally favourable performance.

HA added directly to proteins could be hydrated during homogenisation using water molecules dispersed in the matrix. After denaturation, some of the protein-bound proteins were released and immediately captured by the HA present in the colloidal space. In turn, HA hydrated before being added to the matrix may not have been able to capture more water and was dispersed unevenly in the form of a hydrocolloid, which was confirmed by SEM analyses (Figure 3). When HA is pre-dissolved in water, it rapidly adopts an expanded hydrated conformation, which may lead to localised HA-rich domains upon subsequent protein addition. This premature hydration can limit synchronised HA-MP interactions during thermal gelation, resulting in a less uniform protein–polysaccharide network compared to the simultaneous addition approach. In contrast, adding HA directly to the protein matrix may lead to steric hindrance and competition for water, which can impede its uniform dispersion.

Compared to the sequential pre-dissolution approach, the simultaneous addition method resulted in a more homogeneous and interconnected protein network, as evidenced by SEM observations, along with improved viscoelastic properties and enhanced thermal stability. In contrast, although pre-dissolved HA promoted higher water retention in W15HA800MP, it likely led to localised HA-rich domains and less uniform protein–polysaccharide integration, which adversely affected gel structure and mechanical integrity.

DSC and SEM analyses also indicated that HA influenced the organisation of the gel matrix, resulting in a more compact and uniform protein structure. These effects are probably associated with interactions between HA and MP, which contribute to improved gel formation and water retention.

In this study, it was additionally demonstrated that the order of HA incorporation significantly affected the physicochemical and structural properties of MP gels.

What is more, in this study, we explored the impact of the order of HA incorporation on the properties of MP gels. The results revealed that the method of mixing HA with other ingredients significantly influences the texture, ability to hold water and structure of the gels. Among all tested formulations, the MP800HA30W sample—in which HA, water and MP were mixed simultaneously—exhibited the most favourable overall characteristics. It demonstrated the lowest hardness and chewiness while also being highly cohesive and effective in retaining water. These attributes suggest that it forms a well-balanced and functional gel, making it particularly suitable for meat products. On the other hand, the W15HA800MP sample—in which HA was pre-dissolved in water before adding the protein—exhibited the highest cooking yield; however, it lacked the desirable texture and structure observed in MP800HA30W. The compact and uniform structure of MP800HA30W indicates the formation of a homogeneous and functionally effective protein–polysaccharide network, which contributes to improved water retention and gel stability, rather than simply increasing gel rigidity.

## 3. Materials and Methods

### 3.1. Ingredients

Pork meat (*m. quadriceps femoris*) was purchased from a local retail shop (Macro Cash and Carry, Kraków, Poland) and used as the primary protein source. Food-grade sodium hyaluronate (94.27% HA, molecular weight: 1.27 MDa) was supplied by Beijing Wisapple Biotech Co., Ltd., Beijing, China. It was made by fermenting bacteria with *Streptococcus zooepidermicus.*

### 3.2. Extraction of Myofibrillar Proteins

MP was extracted using the method proposed by Cao et al. [43], with slight modifications. In summary, we ground the pork meat using a meat grinder (MADO MEW 613 grinder, Dronhan, Germany), with a Ø3 mm plate. Then, four volumes of buffer I (pH of 7.0, 10 mmol·L^−3^ Na_2_HPO_4_, 2 mmol·L^−3^ MgCl_2_, 1 mmol·L^−3^ EGTA and 0.5 mmol·L^−3^ DTT) were added to the meat. The mixing was carried out three times, with 10,000 rpm each time. After the mixture was homogenised, it was centrifuged (Thermo Fisher Scientific, Karlsruhe, Germany) at 3000× *g* for 15 min at 4 °C, and the supernatant was removed. This step was repeated twice using buffer I. Subsequently, the sediments were mixed again in four volumes of buffer II (0.1 mol·L^−2^ NaCl, 1 mmol pH 6.25, 4 °C) and filtered through a single layer of gauze. The homogenates were centrifuged once more at 3000× *g* for 20 min at 4 °C, and the sediments were collected. This step was repeated twice using buffer II, with filtration through gauze at each step. The proteins were collected after six cycles of centrifugation, and the Biuret method was used to measure the protein concentrations. Bovine serum albumin (BSA; Thermo Scientific™, Pierce™, Rockford, IL, USA) was used as a standard. The proteins were used within 48 h.

### 3.3. Sample Preparation

To distinguish the effect of the HA addition sequence, two mixing strategies were used. In the simultaneous (combined) addition approach, MP, HA and water were mixed together from the beginning of sample preparation. In the sequential (pre-dissolution) approach, HA was first dissolved in water, followed by the addition of MP. These two preparation methods were applied to examine how the relative timing of HA hydration and MP network formation affects protein-polysaccharide interactions and the resulting gel properties. The formulations and corresponding mixing orders are summarised in Table 6.

MPs were prepared and mixed with HA at two concentrations (400 and 800 mg per 100 g MP) and water at two levels (15 and 30 mL per 100 g MP). The samples differed based on the order of mixing. All variants were conducted in triplicate as independent batches.

### 3.4. Rheological Characteristics

The rheological behaviour of the samples containing HA, MP and water was investigated via the RS-6000 rotational rheometer (Thermo Fisher, Karlsruhe, Germany) using a parallel plate (0.519 mm gap, 35 mm diameter) geometry probe at 20 °C. Before analysis, the samples were conditioned at room temperature for 20 min. During the tests, the material was protected with a plastic cover to reduce moisture evaporation. The stress sweep tests were performed in oscillation mode for stress sweeps at a frequency of 1 Hz and a strain amplitude ranging from 0.01 to 1.00. We recorded the yield stress of the tested materials as the intersection of the G′ and G′ curves. The frequency stress was measured with an applied strain of 0.015, falling within the linear viscoelastic region. The frequencies ranged from 0.1 to 100. All samples were tested in triplicate.

### 3.5. Thermal Analysis via Differential Scanning Calorimetry

We measured the thermal characteristics of the cooked samples using a differential scanning calorimeter—DSC 204F1 Phoenix (Netzsch, Waldkraiburg, Germany). We calibrated the instrument using a multipoint method (Hg, In, Sn, Bi, Zn and CsCl) before conducting the analyses. Approximately 15 mg of material was hermetically closed in aluminium pans and analysed using the DSC temperature programme adjusted between −80 and 200 °C, with a heat rate of 5 °C/min. An empty aluminium pan was used as a reference sample. As a carrier gas agent, nitrogen was used. All analyses were conducted in triplicate. We determined the thermal effects and constants on thermograms using Proteus Analysis software (SW/DSC/670.F1.140; Netzsch, Waldkraiburg, Germany). The onset temperature (To), peak temperature (Tp) and transition enthalpy (H) values were calculated for two main endothermic events.

### 3.6. Determination of Physical Properties for MP-HA Gels

#### 3.6.1. Cooking Yield and pH Determination

Cooking yield was calculated by comparing the weight of the gels before and after heating them at 100 °C for 20 min, following the general procedure described by Liu et al. (2015) [44]. Surface moisture was blotted off prior to weighing.

$$\begin{array}{rr} & Cooking\;Yield=\frac{Cooked\;Weight}{Raw\;Weight}\times 100 \end{array}$$

Five grams of each sample were mixed with 5 mL of distilled water, and the pH of gels was measured using the Elmetron CP-505 pH metre (Zabrze, Poland).

#### 3.6.2. Colour Determination

A colourimeter (CR-400, Konica Minolta Sensing INC., Tokyo, Japan) was employed to assess the colour of the cooked samples using an 8 mm aperture, D illuminant and 10° observation angle. The colourimeter was calibrated before measurement using two calibration standards: a white and a black calibration plate, ensuring accurate and consistent colour readings. The colour was expressed in terms of L* (lightness), a* (green-red) and b* (blue-yellow) values, measured in six replicates, and the average values were calculated. In addition, the whiteness index (WI) and yellowness index (YI) were determined as described by Stępień et al. [45], and using the following equations:

$$\begin{array}{ll} & WI=100-\sqrt{{\left(100-L^{*}\right)}^{2}+{\left(a^{*}\right)}^{2}+{\left(b^{*}\right)}^{2}}\\ & YI=\frac{142.86\times b^{*}}{L^{*}} \end{array}$$

The total colour difference (ΔE) between samples was calculated according to the CIE 1976 formula, where L*_1_, a*_1_ and b*_1_ are the CIELAB values of the control sample and L*_2-13_, a*_2-13_, b*_2-13_ are those of the treated samples.

$$\Delta E^{*}=\sqrt{{\left(L_{1}^{*}-L_{2}^{*}\right)}^{2}+{\left(a_{1}^{*}-a_{2}^{*}\right)}^{2}+{\left(b_{1}^{*}-b_{2}^{*}\right)}^{2}}$$

This method was adopted following Hernández Salueña et al. (2019) for meat colour analysis during shelf-life [46].

#### 3.6.3. Texture Analysis

The textural properties were evaluated using a texture analyser (TA. XT. Plus, Stable Microsystems, Surrey, UK) equipped with the P100 probe. Cooked samples were shaped into cylinders (1.5 cm in diameter, 1.5 cm in height) and tested at pre-test, test and post-test speeds of 5 mm/s, 3 mm/s and 5 mm/s, respectively, with a strain level of 50% and an auto force of 5 g. The measured parameters included hardness, adhesiveness, springiness, cohesiveness, chewiness and resilience.

#### 3.6.4. Scanning Electron Microscopy (SEM) Analysis

The microstructure of the cooked samples was examined using the Quattro ESEM microscope (Thermo Fisher Scientific) equipped with an FEG (field emission gun) electron source. The samples were freeze-dried and cut into 4 × 4 × 4 mm cubes from the central part of each sample using a scalpel. The sections were mounted on pin stubs using pre-mounted carbon discs and imaged without an additional conductive coating. Imaging was performed at an accelerating voltage of 20 kV, with a magnification of 400× and in low vacuum conditions (200 Pa, spot size 4.0) to minimise structural alterations. A LVD (SE) detector was used for image acquisition to reduce artefacts caused by rapid drying in the microscope chamber. The working distance was set to 10.1 mm, and the horizontal field width was 1.04 mm. SEM images were quantitatively analysed using ImageJ (online version, https://ij.imjoy.io). Images were calibrated using a scale bar (50 µm), converted to 8-bit grayscale, and thresholded to distinguish protein networks from void spaces. The area fraction of voids (porosity) and average pore size were measured to compare network density and homogeneity between samples.

### 3.7. Statistical Analysis

All statistical analyses were performed using R software within the RStudio environment (version 4.4.2; RStudio, PBC, Boston, MA, USA). Analysis of variance (ANOVA) was utilised to assess the effects of HA and water levels on the textural and colour parameters of myofibrillar protein gels. Yield data showed heterogeneity of variances; therefore, Welch’s one-way ANOVA was applied, followed by post hoc comparisons using the Games–Howell test, which is suitable for unequal variances and sample sizes. For colour and textural parameters, assumptions of normality and homogeneity of variances were fulfilled; thus, standard one-way ANOVA was used, followed by Tukey’s Honestly Significant Difference (HSD) test for multiple comparisons (*p* < 0.05). The results are expressed as means ± standard errors (SE), indicating the average values along with their variability. Different letters within the same column indicate significant differences (*p* < 0.05); if no significant differences were observed, the same letter (a) was assigned to all groups. The statistical analyses were performed using the agricolae, dplyr and tidyverse packages in R, chosen for their capabilities in handling agricultural experiments and data manipulation.

## 4. Conclusions

In conclusion, these findings highlight that mixing HA with water and protein simultaneously leads to the formation of gels with superior functional qualities. Therefore, optimising the order of HA incorporation represents a crucial strategy for enhancing the performance, consumer appeal and industrial applicability of meat-based gels in contemporary food formulations. Importantly, the addition of HA induced only minor changes in pH and colour parameters, which remained within acceptable sensory and technological limits. The results obtained in this study suggest that HA can be effectively utilised, without adversely affecting the visual or physicochemical quality of meat products. Overall, the ability of HA to improve both technological functionality and sensory attributes such as texture and colour highlights its potential utility in the development of high-quality processed meat products.

Beyond the laboratory, the findings offer valuable implications for the meat processing industry, supporting the development of products with improved quality, yield and consumer appeal. As the market demand for healthier and more sustainable food products continues to grow, the incorporation of functional ingredients such as HA can help meet evolving consumer expectations while improving manufacturing efficiency. Based on these findings, future research should be carried out to explore sensory evaluations on consumer acceptance of HA-enriched meat products. Additionally, examining the long-term storage stability of these products in various conditions would provide valuable information regarding their commercial viability. Further investigation into the optimal concentration of HA and its interactions with other hydrocolloids could lead to more effective formulations. Moreover, extending the application of HA to various meats and protein-based systems may foster innovation across the food industry.

When combined, these results show that HA can be used successfully in meat products, such as sausages, as a natural substitute for traditional water-binding agents or texture enhancers. In addition to its functional qualities, HA is a naturally occurring biopolymer with established physiological advantages, which may add to the finished product’s potential to promote health.

From a process-engineering perspective, in our study, it is indicated that the order of HA addition can be seen as a critical design parameter with direct influence on formulation variables and structure function properties such as WHC, gel strength and network homogeneity. The improved performance of the co-addition method (HA + water + MP) provides an industrially applicable operating window that can be integrated into commercial mixing and hydration protocols. Our results are transferable to other protein-based gels, and they offer scope for scale-up, process optimisation and consistent quality in commercial meat processing.

## Figures and Tables

**Table 1:** Thermal parameters values of hyaluronic acid—myofibrillar proteins—water systems obtained by DSC (mean ± SE).

Sample	Endothermic Peak 1—Melting			Endothermic Peak 2—Thermal Degradation		
	To [°C]	Tp [°C]	ΔH [J/g]	To [°C]	Tp [°C]	ΔH [J/g]
MP	−2.20 ^a^ ± 0.00	11.73 ^b^ ± 0.27	211.83 ^b^ ± 3.01	157.90 ^b^ ± 1.76	173.23 ^a^ ± 2.23	1674.33 ^a^ ± 52.42
MP15W	−1.60 ^a^ ± 0.10	13.97 ^a^ ± 0.63	187.60 ^c^ ± 0.70	162.43 ^a^ ± 2.07	167.10 ^a^ ± 2.90	938.80 ^c^ ± 8.24
MP30W	−1.50 ^a^ ± 0.23	9.93 ^b^ ± 0.73	360.47 ^a^ ± 6.03	156.50 ^b^ ± 3.78	163.60 ^a^ ± 1.26	2025.27 ^a^ ± 24.55
MP400HA15W	−0.97 ^b^ ± 0.27	11.77 ^a^ ± 1.09	233.30 ^b^ ± 14.53	136.03 ^c^ ± 1.70	155.90 ^b^ ± 6.40	1407.00 ^b^ ± 47.35
MP400HA30W	−1.33 ^b^ ± 0.37	12.90 ^a^ ± 0.73	269.27 ^b^ ± 7.87	133.10 ^c^ ± 3.11	139.93 ^c^ ± 5.77	353.60 ^d^ ± 21.95
W15HA400MP	−1.47 ^a^ ± 0.12	10.73 ^b^ ± 0.59	268.20 ^b^ ± 4.21	145.37 ^b^ ± 4.18	151.87 ^b^ ± 2.48	1583.67 ^a^ ± 11.13
W30HA400MP	−1.77 ^a^ ± 0.29	10.73 ^b^ ± 0.06	118.20 ^d^ ± 0.64	166.63 ^a^ ± 0.66	174.30 ^a^ ± 0.50	502.73 ^c^ ± 1.51
MP800HA	−1.93 ^a^ ± 0.07	10.03 ^b^ ± 0.17	230.03 ^b^ ± 4.97	158.87 ^b^ ± 1.36	166.40 ^a^ ± 4.10	1324.67 ^b^ ± 28.35
MP800HA15W	−1.60 ^a^ ± 0.53	11.87 ^a^ ± 0.99	195.56 ^c^ ± 4.01	136.00 ^c^ ± 2.20	142.30 ^c^ ± 5.40	346.73 ^d^ ± 35.97
MP800HA30W	−1.80 ^a^ ± 0.21	10.70 ^b^ ± 0.15	273.97 ^b^ ± 11.05	160.83 ^a^ ± 4.91	167.00 ^a^ ± 1.62	317.20 ^d^ ± 23.50
W15HA800MP	−1.47 ^a^ ± 0.07	10.07 ^b^ ± 0.53	235.10 ^b^ ± 2.05	158.70 ^b^ ± 0.10	160.90 ^b^ ± 0.55	706.47 ^c^ ± 10.36
W30HA800MP	−0.57 ^b^ ± 0.20	12.03 ^a^ ± 0.53	233.77 ^b^ ± 0.38	144.70 ^b^ ± 2.50	147.57 ^b^ ± 0.94	292.60 ^d^ ± 15.24

a, b, c, d—different letters in superscript indicate significant differences between means in same column at *p* < 0.05.

**Table 2:** Effects of hyaluronic acid and water levels on cooking yield and pH of meat protein gels (mean ± SE).

Sample	Yields	pH [ns]
MP	71.6 ^ab^ ± 1.3	6.98 ± 0.00
MP15W	66.7 ^ab^ ± 2.3	6.97 ± 0.01
MP30W	61.2 ^a^ ± 2.4	6.98 ± 0.00
MP400HA	79.7 ^ab^ ± 3.6	6.89 ± 0.03
MP400HA15W	79.4 ^ab^ ± 6.2	6.90 ± 0.03
MP400HA30W	74.3 ^ab^ ± 7.2	6.90 ± 0.02
W15HA400MP	78.2 ^ab^ ± 2.5	6.85 ± 0.04
W30HA400MP	77.4 ^ab^ ± 1.7	6.86 ± 0.04
MP800HA	81.3 ^ab^ ± 5.9	6.89 ± 0.04
MP800HA15W	80.9 ^ab^ ± 5.5	6.92 ± 0.02
MP800HA30W	77.2 ^ab^ ± 6.1	6.89 ± 0.02
W15HA800MP	82.3 ^b^ ± 2.4	6.90 ± 0.03
W30HA800MP	79.7 ^ab^ ± 3.2	6.88 ± 0.02

a, b—different letters in superscript indicate significant differences between means in same column at *p* < 0.05. [ns]—no significant differences were detected.

**Table 3:** Effects of hyaluronic acid and water levels on colour parameters (L*, a*, b*, WI, YI) of myofibrillar protein gels (mean ± SE).

Samples	L* [ns]	a*	b*	WI [ns]	YI	ΔE
MP	74.16 ± 0.30	−1.53 ^ab^ ± 0.01	5.77 ^ab^ ± 0.09	73.48 ± 0.31	11.13 ^ab^ ± 0.21	-
MP15W	74.69 ± 0.28	−1.51 ^a^ ± 0.02	5.53 ^abc^ ± 0.10	74.05 ± 0.29	10.59 ^ab^ ± 0.22	0.58
MP30W	74.87 ± 0.24	−1.51 ^a^ ± 0.02	5.44 ^abc^ ± 0.10	74.25 ± 0.25	10.39 ^ab^ ± 0.22	0.78
MP400HA	74.47 ± 0.27	−1.65 ^bcd^ ± 0.02	5.96 ^a^ ± 0.15	73.73 ± 0.30	11.45 ^a^ ± 0.33	0.38
MP400HA15W	73.89 ± 0.53	−1.71 ^cd^ ± 0.04	5.52 ^abc^ ± 0.10	73.26 ± 0.53	10.69 ^ab^ ± 0.26	0.41
MP400HA30W	74.77 ± 0.25	−1.71 ^cd^ ± 0.04	5.21 ^bc^ ± 0.15	74.18 ± 0.27	9.97 ^b^ ± 0.31	0.84
W15HA400MP	74.82 ± 0.25	−1.59 ^abc^ ± 0.02	5.57 ^abc^ ± 0.10	74.16 ± 0.26	10.65 ^ab^ ± 0.21	0.69
W30HA400MP	74.91 ± 0.31	−1.62 ^abc^ ± 0.02	5.22 ^bc^ ± 0.15	74.32 ± 0.33	9.98 ^b^ ± 0.32	0.93
MP800HA	74.37 ± 0.11	−1.69 ^cd^ ± 0.03	5.88 ^a^ ± 0.12	73.64 ± 0.13	11.3 ^a^ ± 0.24	0.28
MP800HA15W	74.37 ± 0.12	−1.70 ^cd^ ± 0.03	5.74 ^abc^ ± 0.12	73.68 ± 0.14	11.03 ^ab^ ± 0.23	0.27
MP800HA30W	74.56 ± 0.41	−1.78 ^d^ ± 0.05	5.43 ^abc^ ± 0.12	73.92 ± 0.42	10.42 ^ab^ ± 0.28	0.58
W15HA800MP	74.81 ± 0.33	−1.64 ^abc^ ± 0.03	5.48 ^abc^ ± 0.10	74.17 ± 0.33	10.48 ^ab^ ± 0.22	0.72
W30HA800MP	74.59 ± 0.30	−1.65 ^bcd^ ± 0.01	5.18 ^c^ ± 0.14	74.01 ± 0.31	9.94 ^b^ ± 0.30	0.74

a, b, c, d—different letters in superscript indicate significant differences between means in same column at *p* < 0.05. [ns]—no significant differences were detected.

**Table 4:** Effects of hyaluronic acid and water levels on textural properties of myofibrillar protein gels (mean ± SE).

Samples	Hardness [N]	Adhesiveness	Springiness	Cohesiveness [ns]	Chewiness	Resilience [ns]
MP	23.26 ^a^ ± 1.90	−9.90 ^a^ ± 1.91	0.91 ^a^ ± 0.02	0.55 ± 0.05	11.58 ^a^ ± 1.26	0.21 ± 0.02
MP15W	14.64 ^bc^ ± 0.66	−17.57 ^ab^ ± 2.29	0.87 ^a^ ± 0.03	0.51 ± 0.04	6.54 ^bc^ ± 0.54	0.20 ± 0.03
MP30W	12.04 ^cdef^ ± 0.44	−37.95 ^abcd^ ± 8.54	0.77 ^ab^ ± 0.10	0.51 ± 0.06	4.53 ^cde^ ± 0.39	0.20 ± 0.04
MP400HA	20.55 ^ab^ ± 1.89	−38.78 ^bcd^ ± 10.54	0.81 ^a^ ± 0.02	0.53 ± 0.02	8.95 ^ab^ ± 1.20	0.20 ± 0.01
MP400HA15W	9.49 ^cdefg^ ± 0.99	−21.49 ^ab^ ± 3.88	0.71 ^ab^ ± 0.11	0.51 ± 0.08	3.15 ^cde^ ± 0.45	0.19 ± 0.05
MP400HA30W	6.58 ^defg^ ± 0.46	−22.38 ^ab^ ± 3.76	0.61 ^ab^ ± 0.09	0.52 ± 0.11	1.94 ^de^ ± 0.30	0.21 ± 0.06
W15HA400MP	13.24 ^cd^ ± 1.22	−30.91 ^abc^ ± 1.06	0.83 ^a^ ± 0.00	0.40 ± 0.01	4.43 ^cde^ ± 0.43	0.14 ± 0.01
W30HA400MP	8.79 ^cdefg^ ± 1.55	−33.25 ^abc^ ± 2.10	0.78 ^ab^ ± 0.06	0.34 ± 0.02	2.46 ^de^ ± 0.71	0.10 ± 0.01
MP800HA	12.10 ^cde^ ± 3.24	−27.91 ^ab^ ± 5.00	0.64 ^ab^ ± 0.13	0.58 ± 0.04	4.86 ^cd^ ± 1.80	0.22 ± 0.03
MP800HA15W	5.19 ^efg^ ± 0.91	−60.04 ^cd^ ± 6.69	0.63 ^ab^ ± 0.05	0.34 ± 0.03	1.16 ^de^ ± 0.30	0.09 ± 0.01
MP800HA30W	3.49 ^g^ ± 0.53	−44.12 ^bcd^ ± 6.17	0.61 ^ab^ ± 0.05	0.40 ± 0.06	0.82 ^e^ ± 0.13	0.11 ± 0.03
W15HA800MP	4.82 ^fg^ ± 0.48	−20.03 ^ab^ ± 0.92	0.41 ^b^ ± 0.05	0.69 ± 0.13	1.34 ^de^ ± 0.27	0.30 ± 0.08
W30HA800MP	5.01 ^efg^ ± 1.31	−65.88 ^d^ ± 11.03	0.59 ^ab^ ± 0.11	0.43 ± 0.11	1.15 ^de^ ± 0.34	0.15 ± 0.06

a, b, c, d, e, f, g—different letters in superscript indicate significant differences between means in same column at *p* < 0.05. [ns]—no significant differences were detected.

**Table 5:** Quantitative microstructural parameters of MP gels determined by ImageJ analysis of SEM images.

Samples	Count	Total Area (µm^2^)	Average Size (µm^2^)	%Area
MP	4122	9547.68	2.316	20.056
MP15W	3717	9305.428	2.503	19.539
MP30W	3760	3136.073	0.834	6.455
MP400HA	3785	8564.198	2.263	17.862
MP400HA15W	3898	872.389	0.224	6.397
MP400HA30W	4123	3177.271	0.771	6.672
W15HA400MP	3953	13,755.711	3.48	28.883
W30HA400MP	3146	8341.666	2.652	17.515
MP800HA	2653	9566.953	3.606	20.09
MP800HA15W	6173	2612.183	0.423	18.947
MP800HA30W	5546	3898.477	0.486	19.901
W15HA800MP	4050	1613.33	0.398	11.958
W30HA800MP	3975	3610.701	0.908	26.189

**Table 6:** Experimental sample combinations with different hyaluronic acid (HA) concentrations and water contents with 100 g of myofibrillar proteins (MP).

Sample Code		Mixing Order	Hyaluronic Acid (mg)	Water (mL)
1	MP			0
2	MP15W			15
3	MP30W		0	30
4	MP400HA			0
5	MP400HA15W	Combined		15
6	MP400HA30W			30
7	W15HA400MP		400	15
8	W30HA400MP	(HA + H_2_O) + MP		30
9	MP800HA			0
10	MP800HA15W	Combined		15
11	MP800HA30W			30
12	W15HA800MP		800	15
13	W30HA800MP	(HA + H_2_O) + MP		30

## Data Availability

The original contributions presented in this study are included in the article. Further inquiries can be directed to the corresponding author.

## Abbreviations

The following abbreviations are used in this manuscript:

MP	Myofibrillar Proteins
HA	Hyaluronic acid
EU	European Union
KC	k-carrageenan
SEM	Scanning Electron Microscopy
MHC	Myosin Heavy Chain
DSC	Differential Scanning Calorimetry
SH	Sodium Hyaluronate
WPI	Whey Protein Isolate
WPH	Whey Protein Hydrolysate
WI	Whiteness Index
YI	Yellowness Index
